# Effect of active smoking on the human bronchial epithelium transcriptome

**DOI:** 10.1186/1471-2164-8-297

**Published:** 2007-08-29

**Authors:** Raj Chari, Kim M Lonergan, Raymond T Ng, Calum MacAulay, Wan L Lam, Stephen Lam

**Affiliations:** 1 Department of Cancer Genetics and Developmental Biology, British Columbia Cancer Research Centre, Vancouver, BC, Canada; 2 Department of Computer Science, University of British Columbia, Vancouver, BC, Canada; 3 Department of Cancer Imaging, British Columbia Cancer Research Centre, Vancouver, BC, Canada

## Abstract

**Background:**

Lung cancer is the most common cause of cancer-related deaths. Tobacco smoke exposure is the strongest aetiological factor associated with lung cancer. In this study, using serial analysis of gene expression (SAGE), we comprehensively examined the effect of active smoking by comparing the transcriptomes of clinical specimens obtained from current, former and never smokers, and identified genes showing both reversible and irreversible expression changes upon smoking cessation.

**Results:**

Twenty-four SAGE profiles of the bronchial epithelium of eight current, twelve former and four never smokers were generated and analyzed. In total, 3,111,471 SAGE tags representing over 110 thousand potentially unique transcripts were generated, comprising the largest human SAGE study to date. We identified 1,733 constitutively expressed genes in current, former and never smoker transcriptomes. We have also identified both reversible and irreversible gene expression changes upon cessation of smoking; reversible changes were frequently associated with either xenobiotic metabolism, nucleotide metabolism or mucus secretion. Increased expression of *TFF3*, *CABYR*, and *ENTPD8 *were found to be reversible upon smoking cessation. Expression of *GSK3B*, which regulates *COX2 *expression, was irreversibly decreased. *MUC5AC *expression was only partially reversed. Validation of select genes was performed using quantitative RT-PCR on a secondary cohort of nine current smokers, seven former smokers and six never smokers.

**Conclusion:**

Expression levels of some of the genes related to tobacco smoking return to levels similar to never smokers upon cessation of smoking, while expression of others appears to be permanently altered despite prolonged smoking cessation. These irreversible changes may account for the persistent lung cancer risk despite smoking cessation.

## Background

Lung cancer has the highest mortality rate among all types of malignancies, accounting for approximately 29% of all cancer-related deaths in the United States [1]. It has been estimated that in 2006 alone, the number of new lung cancer cases will exceed 174,000 and approximately 163,000 people will die of this disease [1]. Tobacco smoking accounts for 85% of the lung cancers. Former heavy smokers remain at an elevated risk for developing lung cancer even years after they stop smoking [2,3]. Fifty percent of newly diagnosed lung cancer patients are former smokers [4]. It is therefore important to understand the effects of tobacco smoking on the bronchial epithelium in both active and former smokers.

Recently, a large-scale microarray study characterized gene expression differences between current, former, and never smokers [5], and identified specific genes related to xenobiotic functions, anti-oxidation, cell adhesion and electron transport to be more highly expressed in current smokers relative to never smokers. Genetic regulators of inflammation and putative tumor suppressor genes exhibited decreased expression in current smokers relative to never smokers. Most significantly, a number of genes were identified that exhibited irreversible expression changes upon smoking cessation.

Additional reports have also identified increased expression of various xenobiotic metabolic enzymes including members of the cytochrome P450 (CYP) and glutathione S-transferase (GST) families of proteins in response to cigarette smoke exposure [5-10]. CYP enzymes mediate the conversion of *benzo (a) pyrene *and other polycyclic aromatic hydrocarbons (PAH) to carcinogenic intermediates that interact with genomic DNA [8], thus contributing to the formation of DNA adducts in smokers [11-13]. Members from both of the *CYP *and *GST *gene families have been implicated as potential susceptibility loci mediated by the presence of single nucleotide polymorphisms (SNPs) leading to aberrant expression in response to smoking [14,15].

Another important process associated with tobacco smoke exposure is the airway mucosal response. In animal models, it has been shown that exposure to cigarette smoke induces goblet cell hyperplasia with accompanied mucus production [16,17]. Moreover, mucin 5 (MUC5AC), has been shown to be the most highly expressed mucin in bronchial secretions [18], induced in response to cigarette smoke through an EGFR-dependent mechanism [19]. However, beyond this, little is known of the genes that are associated with airway remodeling as a result of tobacco smoking.

Serial analysis of gene expression (SAGE) is a quantitative experimental procedure widely used to determine expression profiles through the enumeration of short sequence tags and their relative abundance [20]. Although the construction and sequencing of an individual SAGE library is expensive and laborious compared to microarray analysis, SAGE offers the invaluable potential for gene discovery as the analysis is not limited to genes represented on an array. Moreover, comparisons between independent experiments can be performed without sophisticated normalization [21,22].

In this study, we compare the bronchial epithelial transcriptomes of current, former, and never smokers to determine the effect of active smoking on gene expression using bronchial brushings from the peripheral sub-segmental airways. Genes whose expression is reversible upon smoking cessation are expected to differ in abundance between current and former smokers, but are similar between former and never smokers. Conversely, gene expression that is irreversible upon smoking cessation will show similar levels in current and former (ever) smokers but differ between ever and never smokers. Here, we focus on identifying both reversible and irreversible gene expression changes and specifically consider these expression changes in the context of airway mucosal response, and susceptibility to cancer development.

## Results and Discussion

### SAGE library statistics

**Figure 1:**
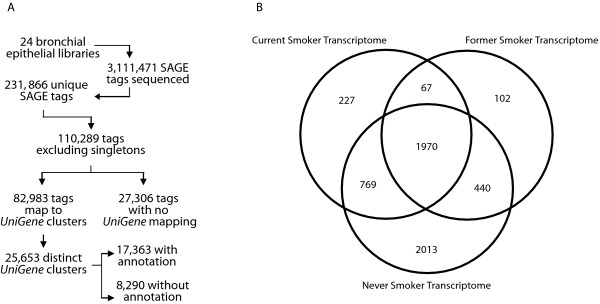
(A) SAGE library statistics: Summary statistics of the 24 SAGE libraries analyzed in this study. Mapping information was based on the May 10th, 2006 version of *SAGEGenie *[45]. In total, over 3,000,000 SAGE tags were sequenced, with over 110,000 unique tags represented upon the exclusion of super singleton tags. (Super singleton tags are tags which have a count of 1 in a single library only). Approximately 75 % of these 110,000 unique tags, (potentially representing as many unique transcripts), mapped to an annotated *UniGene *cluster. As multiple SAGE tags frequently map to the same *UniGene *cluster, we have identified at a total of 25,653 distinct *UniGene *clusters within our dataset, approximately 68% of which represent previously characterized genes. Notably, 25% of the unique tags had no mapping, suggesting much information is currently unknown. (B) Transcriptome Venn diagram: Venn diagram of the transcriptomes of current, former and never smokers. Reported is the number of tags which are expressed in every library group at a raw tag count greater than or equal to 2, representing the tags which are constitutively expressed in each set. Nearly 2000 SAGE tags, mapping to over 1700 genes are common to all 24 SAGE libraries. A lower number of never smokers may have contributed to a higher number of preferentially expressed transcripts in this group.

Twenty-four SAGE libraries were constructed from bronchial epithelial specimens acquired from eight current smokers, twelve former smokers and four never smokers (Table 1). A former smoker was defined as someone who had stopped smoking for one year or longer. The smoking status was verified using exhaled carbon monoxide monitoring. Raw SAGE data for these transcriptomes has been made publicly available at National Center for Biotechnology Information (NCBI) Gene Expression Omnibus (GEO) with series accession number GSE5473. From these 24 libraries, we have collectively sequenced 3,111,471 SAGE tags, yielding 231,866 unique tags, making this the largest human SAGE study reported to date (Figure 1A). Of the unique tags, nearly half were present in more than one library at a tag count of one or greater, and 70% (82,983 tags) of these tags map to a *UniGene *cluster. As multiple tags frequently map to the same *UniGene *cluster, 25,653 unique *UniGene *clusters are represented in our dataset. Significantly, over 27,000 tags did not map to existing annotated genes, reiterating the continuing potential of re-mining this large dataset as tag-to-gene mapping improves with the continuing annotation of human transcripts.

**Table 1:** Demographics of subjects in study

**Sample Name**	**Gender**	**Age at Analysis**	**Pack-Years**	Smoking Status*	**Years of smoking cessation (years)**	**Lung function (predicted FEV_1 _%)**	Name from previous study**
Current 1	F	63	40	CS	N/A	69	BE-13
Current 2	M	56	62	CS	N/A	89	BE-7
Current 3	F	63	44	CS	N/A	96	BE-12
Current 4	M	68	81	CS	N/A	76	BE-1
Current 5	M	64	45	CS	N/A	73	BE-2
Current 6	M	66	53	CS	N/A	85	-
Current 7	M	52	48.1	CS	N/A	63	-
Current 8	F	55	34.4	CS	N/A	81	-
Former 1	M	68	33	FS	19	50	BE-3
Former 2	M	69	100	FS	1	21	BE-4A/4B
Former 3	M	68	30	FS	1	30	BE-9
Former 4	M	70	75	FS	17	76	BE-5
Former 5	M	67	55	FS	5	N/A	BE-6
Former 6	M	65	82	FS	10	59	BE-10
Former 7	F	56	64	FS	1.5	71	BE-11A
Former 8	F	63	45	FS	4.5	83	BE-14
Former 9	M	72	40	FS	32	87	BE-15
Former 10	F	71	56	FS	16	58	BE-16
Former 11	M	72	63	FS	6	N/A	BE-8B
Former 12	M	69	55.3	FS	21	57	-
Never 1	M	58	0	NS	N/A	115	-
Never 2	F	56	0	NS	N/A	104	-
Never 3	M	53	0	NS	N/A	N/A	-
Never 4	F	81	0	NS	N/A	N/A	-

* CS = Current Smoker, FS = Former Smoker, NS = Never Smoker

**Subset of samples were used in a previous study by Lonergan *et al *2006 [55]

### Analysis of the current, former and never smoker transcriptomes

We determined both the number of SAGE tags present in each of the current, former and never smoker transcriptomes, as well as those tags equally represented among the three different datasets. The criteria chosen for preferential expression was a threshold of a raw tag count of ≥ 2 across all samples in a particular set, but not existing in the other sets. Out of 3,033 tags expressed in all current smokers, we found 227 preferentially expressed tags (Additional file 1). In former smokers, 102 tags were found to be preferentially expressed (out of 2,579 tags) (Additional file 2), and in never smokers, 2,013 tags were found to be preferentially expressed (out of 5,192) (Additional file 3). It should be noted that the number of tags preferential to the never smoker set is substantially higher, most likely due to the lower sample size of never smokers relative to the other two groups. However, since we are using never smokers as a reference, a larger transcriptome will lessen the likelihood that we would find transcripts that are preferentially expressed in current and former smokers that were not correct. Looking at those tags which are common to all three groups, it was found that 1,970 tags (mapping to 1,733 unique genes) were expressed in all 24 libraries (Additional file 4). A Venn diagram illustrating the expression patterns of these three groups is given in Figure 1B.

### Genes differentially expressed between current and never smokers

We used a Mann Whitney U test to identify tags differentially expressed in the transcriptomes of current and never smokers. Using cut-off requirements of *p *≤ 0.05, and a fold change of the means ≥ 2, we identified 609 SAGE tags (mapping to 487 unique genes) to be differentially expressed between current and never smokers (Additional file 5).

### Supervised clustering and principal component analysis (PCA) of current, former and never smokers

**Figure 2:**
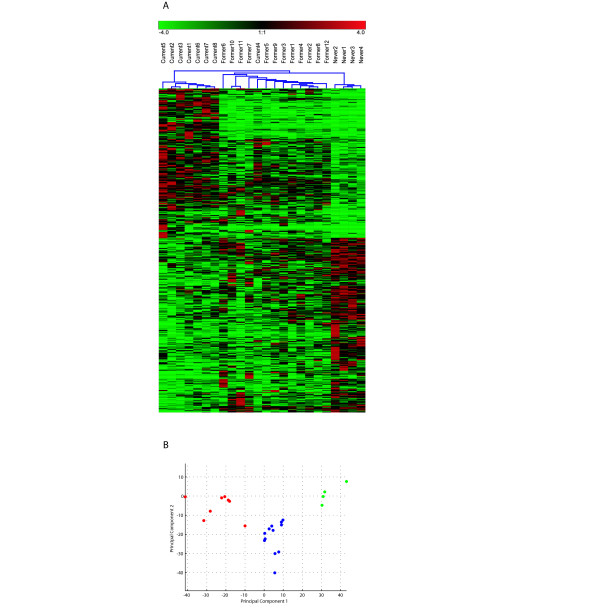
(A) Cluster analysis of current, former and never smokers: Single link hierarchical clustering using the 609 SAGE tags comprised in Additional file 5 representing tags differentially expressed between current and never smokers. Distance measure used was a Euclidean distance. The visualization package *Genesis *[23] was used for clustering. Green rectangles represent samples with lower expression for the particular gene amongst the samples, and red rectangles represent samples where the gene is highly expressed relative to other samples. (B) Principal component analysis of current, former and never smokers. Expression values used were scaled to tags per million (TPM). Each tag was then normalized by dividing its value by the maximum value for that tag seen in all the libraries. Subsequently, this value was then multiplied by 6 and then subtracted by 3 to put the values ratios in the range of -3 to 3. A co-variance based approach was used and the statistics toolbox in *MatLab *(Mathworks) was used. Current smokers are represented in red, former smokers are represented in blue and never smokers are represented in green.

Using the 609 tags found to be differentially expressed between current and never smokers (Additional file 5), single link hierarchical clustering was performed using the program *Genesis *[23]. We hypothesized that these 609 tags would classify current, former and never smokers. Indeed, distinct clusters emerged separating groups of current and former smokers with one exception of Current4 (Figure 2A). Of note, the former smoker who ceased smoking for only one year (Former 2) clustered with other former smokers. Moreover, principal component analysis (PCA) further validates the distinct groups of current, former and never smokers (Figure 2B).

### Reversible gene expression changes upon cessation of smoking

**Figure 3:**
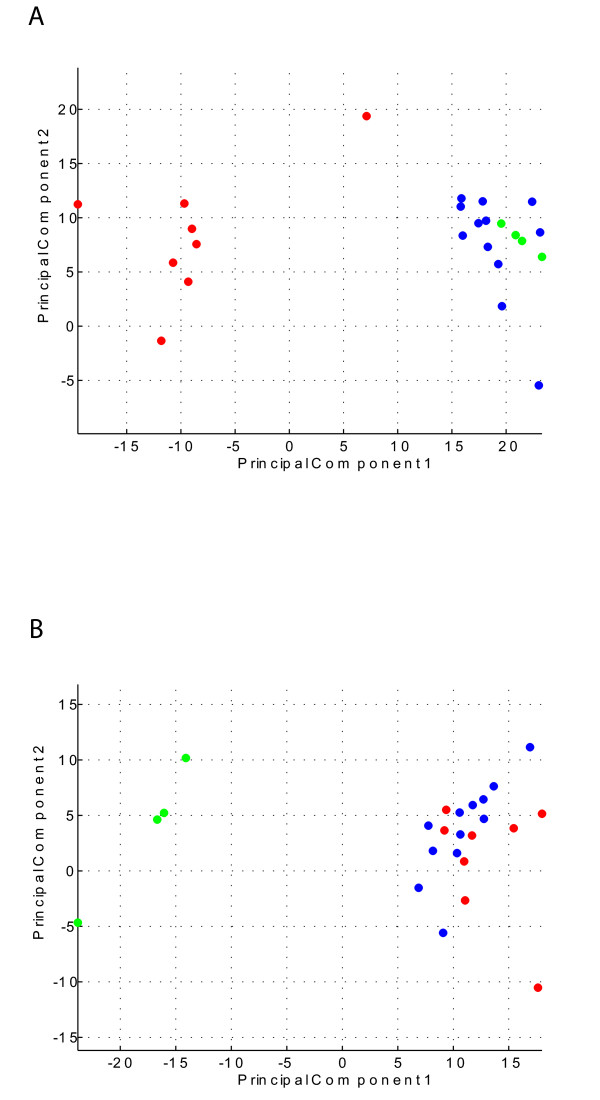
Principal component of current, former and never smokers using (A) the 161 tags deemed reversible upon smoking cessation (Additional file 6) and (B) the 152 tags deemed irreversible upon smoking cessation (Additional file 7). Expression values used were scaled to tags per million (TPM). Each tag was then normalized by dividing its value by the maximum value for that tag seen in all the libraries. Subsequently, this value was then multiplied by 6 and then subtracted by 3 to put the values in the range of -3 to 3. A co-variance based approach was used and the statistics toolbox in *MatLab *(Mathworks) was used. Current smokers are represented in red, former smokers are represented in blue and never smokers are represented in green.

To determine reversibility of smoking-related gene expression changes, we intersected tags differentially expressed between current and never smokers against tags showing significant expression difference between current and former smokers using similar criteria. By comparing these two sets, we can deduce which gene expressions are reversible, i.e., which genes are largely influenced by active smoking. This analysis yielded 161 tags mapping to 121 unique genes, which were deemed statistically significant, and representing 26% of the total number of differentially expressed tags between current and never smokers (Figure 3A, Additional file 6). Further analysis of these 121 differentially expressed genes has identified two main functions: xenobiotic metabolism and nucleotide metabolism (representing 33% of the reversible gene expression changes) (Table 2) and airway mucus secretion (representing 12% of the reversible gene expression changes) (Table 3). Genes related to oxidative stress were considered as part of the xenobiotic metabolism/nucleic acid metabolism category, and those genes previously associated with xenobiotic metabolism and oxidative stress through smoke exposure were among those identified [5,24,25].

**Table 2:** Reversible gene expression upon smoking cessation related to xenobiotic metabolism and DNA adduct formation (genes in bold have not been previously associated with smoking)

**Tag**	**Gene Symbol**	**Gene Name**	CS Mean*	FS Mean*	NS Mean*	**P-Value (CVsF)**
GGCCCAGGCC	ALDH3A1	Aldehyde dehydrogenase 3 family, memberA1	4355	313	261	0.00002
TTAAAAATTC	ADH7	Alcohol dehydrogenase 7 (class IV)	899	145	130	0.00002
AGGTCTGCCA***	AKR1C2	Aldo-keto reductase family 1, member C2	547	116	74	0.00002
AATGCTTTTA	CYP1B1	Cytochrome P450, family 1, subfamily B, polypeptide 1	204	13	0	0.00002
**TTGGAATCCA**	**STAU2**	**Staufen, RNA binding protein, homolog 2 (Drosophila)**	**67**	**17**	**23**	**0.00002**
TTATCAAATC	NQO1	NAD(P)H dehydrogenase, quinone 1	809	202	149	0.00003
CAAATAAACC	PIR	Pirin (iron-binding nuclear protein)	260	47	43	0.00003
GGCCCCATTT	CBR1	Carbonyl reductase 1	144	31	24	0.00003
TATTTTTGTT	TXNRD1	Thioredoxin reductase 1	250	88	78	0.00006
GGTGGTGTCT	GPX2	Glutathione peroxidase 2 (gastrointestinal)	384	40	46	0.00011
**TATTTTTGAA**	**DRB1**	**Developmentally regulated RNA-binding protein 1**	**204**	**32**	**22**	**0.00011**
TGGGAGTGGG**	**NMNAT2**	**Nicotinamide nucleotide adenylyltransferase 2**	**175**	**17**	**9**	**0.00011**
CAAGACCAGT	GSTA2	Glutathione S-transferase A2	1436	485	528	0.00019
GCTTGAATAA	AKR1B10	Aldo-keto reductase family 1, member B10 (aldose reductase)	332	10	15	0.0003
**GTGCAGGGAG**	**SPDEF**	**SAM pointed domain containing ets transcription factor**	**239**	**64**	**50**	**0.0003**
**GGAGGCTTCC**	**MECR**	**Mitochondrial trans-2-enoyl-CoA reductase**	**85**	**26**	**22**	**0.0003**
GTGATGTAAG	SRXN1	Sulfiredoxin 1 homolog (S. cerevisiae)	63	14	11	0.0003
**TATGCTTTAA**	**NT5DC1**	**5'-nucleotidase domain containing 1**	**59**	**23**	**24**	**0.0003**
**GAACGCCTAA**	**DPYSL2**	**Dihydropyrimidinase-like 2**	**1**	**21**	**23**	**0.0004**
TTTTCTGAAA	TXN	Thioredoxin	698	326	212	0.00048
CTTGCATAAG	CYP1A1	Cytochrome P450, family 1, subfamily A, polypeptide 1	89	2	0	0.00048
GCAAGAAGAG	ALDH3A1	Aldehyde dehydrogenase 3 family, memberA1	77	10	2	0.00048
AGAACAAAAC	PRDX1	Peroxiredoxin 1	1043	418	510	0.00071
**AAATATTTAA**	**SLC35A3**	**Solute carrier family 35, member A3**	**47**	**14**	**19**	**0.00071**
CGGCTGAATT	PGD	Phosphogluconate dehydrogenase	252	104	80	0.00106
**CTTATCAGTA**	**BTBD7**	**BTB (POZ) domain containing 7**	**94**	**22**	**2**	**0.00106**
AAGAGTTTTG	AKR1B1	Aldo-keto reductase family 1, member B1 (aldose reductase)	25	5	6	0.00141
GCTGAGATGA**	CYP4F11	Cytochrome P450, family 4, subfamily F, polypeptide 11	22	6	2	0.00141
GGCGCCTCCT	TALDO1	Transaldolase 1	232	76	94	0.00152
**GACACAGCAA**	**ENTPD8**	**Ectonucleoside triphosphate diphosphohydrolase 8**	**24**	**3**	**2**	**0.00159**
**CAGTCTAAAA**	**UCHL1**	**Ubiquitin carboxyl-terminal esterase L1 (ubiquitin thiolesterase)**	**92**	**4**	**0**	**0.00197**
ACATCCTAGG	ALDH1A1	Aldehyde dehydrogenase 1 family, member A1	60	28	30	0.00216
TTAGAAGGAA	NQO1	NAD(P)H dehydrogenase, quinone 1	41	14	9	0.00216
AGGTCTACCA	AKR1C2	Aldo-keto reductase family 1, member C2	270	32	8	0.00292
ATTAGGCCTG	TXNRD1	Thioredoxin reductase 1	51	19	17	0.00297
GAGAGCTTTG	AKR1C3	Aldo-keto reductase family 1, member C3	149	21	22	0.00298
TACGCTTGGT	CYB5R1	Cytochrome b5 reductase 1	68	32	30	0.00298
CACTGCCTTG	FTH1	Ferritin, heavy polypeptide 1	59	23	17	0.00298
CTGCTGCACT	GSR	Glutathione reductase	126	54	50	0.0041
**GGCAAAATTA**	**SLC35A3**	**Solute carrier family 35, member A3**	**73**	**32**	**35**	**0.0041**
ACCTTGGGGT	NQO1	NAD(P)H dehydrogenase, quinone 1	73	19	6	0.0041
**AATGTTCAGG**	**COQ6**	**Coenzyme Q6 homolog, monooxygenase (yeast)**	**29**	**12**	**4**	**0.00708**
**CACTGACCAG**	**NOD27**	**Nucleotide-binding oligomerization domains 27**	**31**	**10**	**0**	**0.00927**
**CTCGGAGGCC**	**SEPX1**	**Selenoprotein X, 1**	**71**	**32**	**28**	**0.00956**
**CTCCAAAAAA**	**CPSF2**	**Cleavage and polyadenylation specific factor 2, 100 kDa**	**118**	**44**	**14**	**0.02013**
AATGGAAACT	GCLM	Glutamate-cysteine ligase, modifier subunit	34	16	9	0.03186

*Mean in tags per million (TPM)

** Changed mapping with *TAGMapper *[56]

***Tag maps with equal reliability to *AKR1C1*

For example, *ectonucleoside triphosphate diphosphohydrolase 8 (ENTPD8)*, an extracellular nucleic acid metabolic enzyme, is among 18 novel genes (labeled in bold in Table II) not previously associated with smoking and whose expression is increased in response to active smoking. According to enzyme classification, *ENTPD8 *is involved in purine and pyrimidine metabolism. Hence, this gene may potentially play a role in the chemical formation of DNA adducts.

Gene expression related to airway muco-ciliary function is also elevated in both current versus former smokers and current versus never smokers (Table 3). For example, *trefoil factor 3 *(*TFF3*), a structural component of mucus that is elevated in inflammatory response [26,27], and *calcium binding tyrosine*-(*Y*) *phosphorylation regulated *(*CABYR*), originally shown to be localized in the principal part of the human sperm flagellum [28], are both highly expressed in current smokers relative to former and never smokers. Though *TFF3 *was recently shown to be expressed in response to chronic exposure of nicotine in intestinal cells [29], this is the first report of this gene being overexpressed within the bronchial epithelium in response to active smoking. Based on its assumed role in sperm motility, *CABYR *may be involved in ciliary function associated with muco-ciliary clearance response within the lung [28]. Interestingly, overexpression of *CABYR *variants have been reported in a variety of brain tumors [30], suggesting a role in carcinogenesis. Previous observation of increased *MUC5AC *expression in current relative to never smokers and increased expression of *microseminoprotein, beta- (MSMB)*, a gene shown to be present in mucosal secretions [31], supports the possibility of induction of airway mucosal response in active smokers [5,24,25,32].

**Table 3:** Reversible gene expression upon smoking cessation related to mucus secretion (genes in bold have not been previously associated with smoking)

**Tag**	**Gene Symbol**	**Gene Name**	CS Mean*	FS Mean*	NS Mean*	**P-Value (CVsF)**
**GAATGAACTG**	**EDIL3**	**EGF-like repeats and discoidin I-like domains 3**	**72**	**5**	**8**	**0.00011**
CTCCACCCGA	TFF3	Trefoil factor 3 (intestinal)	4974	1978	1722	0.00019
**GTGGAGAAGA**	**CLDN10**	**Claudin 10**	**89**	**23**	**26**	**0.00019**
**GGAATTGCCC**	**BPIL1**	**Bactericidal/permeability-increasing protein-like 1**	**43**	**4**	**4**	**0.00029**
TTGGTTTTTG	CXCL6	Chemokine (C-X-C motif) ligand 6	147	414	371	0.0003
CCTATCAGTA	MSMB	Microseminoprotein, beta-	15881	4405	2948	0.00048
**CTTCCTGTGA**	**SBEM**	**Small breast epithelial mucin**	**154**	**27**	**32**	**0.00071**
**TGGAAATGTG**	**CBARA1**	**Calcium binding atopy-related autoantigen 1**	**49**	**18**	**15**	**0.00141**
**CAAGCATAAA**	**CABYR**	**Calcium binding tyrosine-(Y)-phosphorylation regulated**	**63**	**4**	**4**	**0.00241**
AGGGAGGCAG	SCGB1A1	Secretoglobin, family 1A, member 1	135	473	436	0.0041
TATCACATTC	CXCL6	Chemokine (C-X-C motif) ligand 6	10	42	29	0.00573
TTGCACCCTT	MSMB	Microseminoprotein, beta-	71	16	9	0.00735
AGCTTAATGA**	SCGB1A1	Secretoglobin, family 1A, member 1	557	1478	3269	0.00956
GAAAAAATAG	SCGB1A1	Secretoglobin, family 1A, member 1 (uteroglobin)	88	288	281	0.0124
**GTGGCCACGG**	**S100A9**	**S100 calcium binding protein A9 (calgranulin B)**	**26**	**101**	**63**	**0.0124**
**AAAATGTATT**	**CAV2**	**Caveolin 2**	**20**	**6**	**4**	**0.0144**
GACAAGGATG	CX3CL1	Chemokine (C-X3-C motif) ligand 1	29	63	93	0.01586

*Mean in tags per million (TPM)

** Changed mapping with *TAGMapper *[56]

### Irreversible gene expression changes upon cessation of smoking

By intersecting genes which are differentially expressed between current and never smokers with those that are different between former and never smokers, we can identify irreversible gene expression changes upon smoking cessation. This analysis yielded 152 tags (124 unique genes) meeting the criteria of statistical significance (*p *≤ 0.05) at a fold change ≥ 2 (Figure 3B, Additional file 7). Although genes identified by this analysis appear to be functionally diverse, a small number of genes related to the cell cycle process and DNA repair have been identified here. For example, expression of *P21/Cdc42/Rac1-activated kinase 1 (PAK1)*, *cyclin D1 (CCND1)*, and *cyclin G2 (CCNG2) *all appear to be irreversibly lower in ever (former and current) smokers relative to never smokers. This finding is consistent with a previous report of increased inhibition of cell proliferation through genes such as *CDKN1A *in a higher stage (GOLD-2) of chronic obstructive pulmonary disease (COPD) versus the lowest stage (GOLD-0) [33].

We also found genes associated with DNA repair to be differentially expressed between current and never smokers, but similar between current and former smokers. *APEX nuclease (multifunctional DNA repair enzyme) 1 (APEX1)*, *High-mobility group box 1 (HMGB1), REV1-like (REV1L)*, and *Tumor suppressor candidate 4 (TUSC4) *are repair genes which we have found to be irreversibly under-expressed in ever smokers. Significantly, *APEX1 *has been shown to harbor SNPs associated with lung cancer susceptibility [34]. Moreover, *REV1L *is involved with the recruitment of DNA polymerase eta to assist in DNA replication at arrested replication forks in areas of DNA lesions such as those formed by thymine dimmers [35,36]. *TUSC4*, also known as *NPRL2*, has recently been shown to increase sensitivity to cisplatin [37]. Finally, *HMGB1 *has also been suggested to be involved with the recruitment of other repair-related proteins [38].

It should be noted that a significant proportion of former smokers in our sample set exhibited low FEV_1 _levels, raising the possibility that airflow obstruction may be a confounding issue in this analysis. To address this, we used the 20 individuals with available FEV_1 _data to compare individuals with moderate or severe COPD (FEV_1 _< 80%, n = 12) with those individuals that would be classified with at most mild COPD (FEV_1 _≥ 80%, n = 8) according to the GOLD staging classification based on FEV_1 _status [39,40]. Of the 157 tags differentially expressed between these two groups, only 6 tags overlap with our list of irreversible genes (Additional file 8). This minimal overlap suggests that the irreversible genes identified are not significantly associated with airway obstruction based on FEV_1 _status. Nonetheless, airway obstruction should be considered in the interpretation of differential gene expression between current and former smokers.

A similar approach to that described here was undertaken by Spira *et al*. where the expression of 13 genes, including some putative oncogenes and tumor suppressor genes, was deemed irreversible upon cessation of smoking. However, none of these 13 genes overlapped with those identified in our study. This lack of overlap may reflect the differing locations from which the bronchial brushings were obtained as Spira *et al *[5] sampled from the right main bronchus whereas we have sampled peripheral sub-segmental airways.

It is interesting to note that *MUC5AC *appears in both the lists of statistically reversible and irreversible gene expression changes suggesting that expression of this gene exhibits distinct states of expression among current, former and never smokers. Moreover, it should also be noted that although 311 of the 609 tags were classified as either reversible or irreversible, the remaining 298 tags did not meet the statistical criteria for either category.

### Validation of select gene expression changes using quantitative RT-PCR

**Figure 4:**
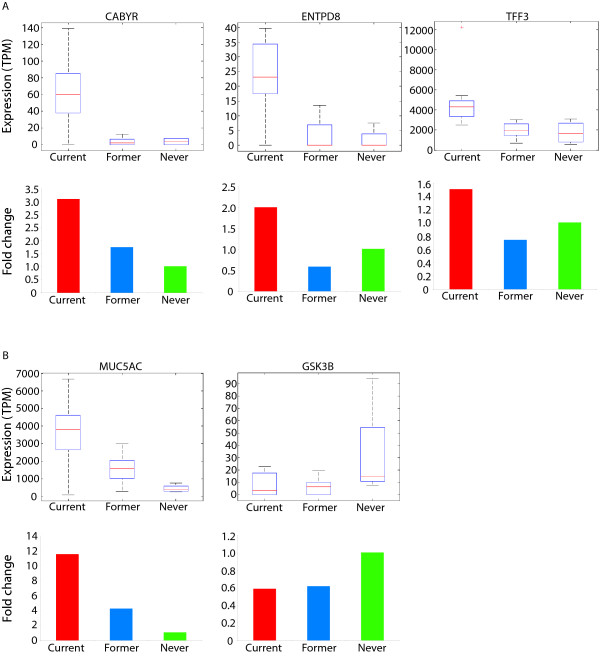
SAGE and quantitative PCR (qRT-PCR) analysis of select genes: (A) Genes found to have reversible expression upon smoking cessation. Box plots of SAGE data and histograms for qRT-PCR for *CABYR, ENTPD8 *and *TFF3*. Distribution of ratios between both current vs. former and current vs. former and never (Additional file IV) were found to be statistically different. (B) Genes found to be either partially or fully irreversible. Box plots of SAGE data and histograms for qRT-PCR for *MUC5AC *and *GSK3B*. Distribution of ratios between current vs. former and former vs. never were statistically different for *MUC5AC *and in addition, *GSK3B *was statistically significant for the combination of current and former vs. never. Box plot analysis was done using the Statistics toolbox from the *MathWorks MatLab *program. Red lines in the boxes represent the median expression value in terms of tags per million (TPM), and red "plus" signs represent outliers (values which are greater than 1.5 times the maximum value). The bottom and top part of the boxes represent the 2^nd ^and 3^rd ^quartiles of the data respectively. The error bars represent the 5^th ^and 95^th ^percentiles of the data. Quantitative RT-PCR validation was performed on a second cohort of nine current smokers, seven former smokers and six never smokers. Plotted is the average expression ratio relative to the average expression in never smokers of current (red), former (blue) and never (green) smokers. Statistical significance was determined using a one-tailed p-value from the Mann Whitney U Test (Supplemental Table IX).

In addition to the SAGE analysis, which identified genes associated with airway mucosal response and xenobiotic/nucleic acid metabolism as distinguishing features between current and former smokers, we have performed quantitative RT-PCR on a secondary cohort of current, former and never smokers to validate selected genes for expression changes (Additional file 9). In total, five genes were selected for validation. From the set of reversible genes, we have chosen *CABYR*, *ENTPD8*, and *TFF3 *because their expression has not been associated with smoking previously. In addition, from the irreversible genes, we have selected *MUC5AC*. Using the delta-delta-Ct method to derive expression values, we then employed a Mann Whitney U Test to determine significance. The pattern of reversible over-expression in current smokers for *CABYR*, *ENTPD8*, and *TFF3 *(Figure 4A) and the irreversible over-expression of *MUC5AC *(Figure 4B) observed from the SAGE data, was validated by quantitative RT-PCR (Additional file 10). Raw cycle thresholds for each gene are available in Additional file 9.

### Airway epithelium response genes and their role in inflammation and cancer

Although the role of xenobiotic metabolism in smoking-induced carcinogenesis has been well documented [9,15], the potential influence mediated by changes in the composition of the airway mucosa in the development of lung cancer, has not been thoroughly investigated. It is possible that constant dysregulation of expression of genes associated with mucus secretion (such as *TFF3 *and *MUC5AC*) by smoking could potentially have a direct or indirect role in smoking-induced carcinogenesis.

One of the many genes involved in lung cancer development is *cyclooxygenase 2 (COX2)*, which plays a multi-faceted role in cellular proliferation, migration and invasiveness [41]. Notably, secretoglobin, family 1A, member 1 (SCGB1A1) protein has been shown to inhibit *COX2 *at the mRNA level [42,43]. We observed that *SCGB1A1 *expression is drastically reduced in current smokers but is expressed at similar levels in former and never smokers, and a previous study showed decreased serum *SCGB1A1 *level in smokers [44]. It should also be noted that none of the SAGE sequence tags identified in the analysis mapping to *SCGB1A1 *are the most reliable tag according to *SAGE Genie *[45]. However, even though the most reliable tag to this gene, CTTTGAGTCC did not pass statistically, the trend of reduced expression in current smokers relative to former smokers and similar expression between former and never smokers is consistent with the sequence tags that did appear in the analysis. Moreover, given that multiple tags have appeared from our analysis, although not as reliably mapped, we are confident that we are detecting *SCGB1A1 *mRNA expression. Interestingly, *COX2 *mRNA expression was not detected in the bronchial epithelium of current, former and never smokers from our SAGE data. A recent report demonstrated a significant increase in *COX2 *expression in normal lung fibroblasts when exposed to cigarette smoke extracts [46]. It is possible that *SCGB1A1 *involvement is in the stroma and not in epithelial cells.

Despite lack of knowledge about *CABYR*, one of its few known interactions occurs with *GSK3B *[30].*CABYR *is a substrate of *GSK3B *[30], and exhibits reversible, increased expression with active smoking (Figure 4A). Though *GSK3B *was not identified as a smoking-related gene in our primary analysis, investigation of the SAGE data revealed a trend of similar decreased expression in current and former smokers relative to never smokers. Moreover, quantitative RT-PCR using a secondary cohort of samples validated that *GSK3B *expression is irreversibly reduced in ever smokers (Figure 4B). Recently, a published report using porcine tracheobronchial epithelial cells exposed to cigarette smoke components *in vitro*, demonstrated an inhibition of *GSK3B *gene expression [47]. *GSK3B *has been shown to negatively interact with *COX2 *[48]. Reduced expression of *GSK3B *may therefore account for exaggerated inflammatory response despite smoking cessation and may contribute to development of lung cancer.

**Figure 5:**
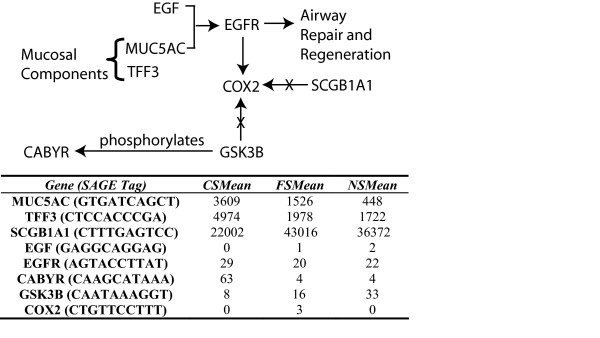
Expression trends of specific genes related to muco-ciliary function and airway restitution as compared with smoking status and lung cancer: *TFF3, CABYR*, and *MUC5AC *are over expressed in current smokers with lowered expression in both former and never smokers. Conversely, *SCGB1A1 *shows the opposite effect, with lowered expression in current smokers as compared to former and never smokers. *MUC5AC *and *TFF3 *are known to be components of mucus. *EGFR *levels are positively correlated with smoking status, with modestly higher levels in current smokers. *MUC5AC *and EGF have been shown to interact with *EGFR *in the process of airway restitution and *SCGB1A1 *has been shown to decrease levels of *cyclooxygenase 2 (COX2) *in cancer cells. Interestingly, within this process alone, we see reversible *(TFF3*, *CABYR)*, partially reversible *(MUC5AC) *and completely irreversible *(GSK3B) *expression changes upon smoking cessation. Values refer to tag counts as tags-per-million (TPM).

In this study, we have demonstrated differential expression of various components of respiratory tract mucus (including *TFF3 *and *MUC5AC*) according to smoking status (Table 3). However, our data indicates that *MUC5AC *expression is not completely reversible upon smoking cessation and in fact, exhibits three statistically distinct levels of expression between current, former and never smokers (Figure 4B). *TFF2*, a related motogen to *TFF3*, in conjunction with *epidermal growth factor (EGF)*, has been shown to promote airway restitution, (i.e., movement of neighboring airway epithelial cells in response to injury mimicking rapid epithelium regeneration), through the activation of the *epidermal growth factor receptor (EGFR) *[49], expressed in the normal bronchial mucosa [50,51]. Other studies have also demonstrated increased expression of *MUC5AC*, along with *EGFR *and *v-erb-b2 erythroblastic leukemia viral oncogene homolog 3 (ERBB3) *in active smokers [26,32]. We examined *EGFR *expression in relation to smoking and found that there was a modest increase of approximately 1.5-fold between current and former smokers in our SAGE data. As enhanced expression of *EGFR *is well documented in lung cancer [52,53], these results imply that enhanced expression of *TFF3 *(and perhaps other genes associated with airway epithelial response and mucus secretion) may promote airway restitution in response to active smoking and that constant induction of airway reconstruction may play a role in the development of lung cancer (Figure 5).

## Conclusion

This study represents the largest human SAGE study reported to date. Over three million SAGE tags were sequenced, representing over 110 thousand potentially unique transcripts expressed within the bronchial epithelium relative to cigarette smoke exposure. These libraries provide a valuable resource for future data mining. Based on the gene expression profiles of 24 current, former and never smokers, we identified both reversible and irreversible gene expression changes upon smoking cessation. Specifically, amongst those genes reversibly expressed, three main functions were identified: xenobiotic metabolism, nucleotide metabolism, and mucus secretion. In addition, some of the genes associated with airway mucosal response are strongly involved with airway epithelium repair and regeneration. Interestingly, investigating airway repair and regeneration revealed genes varying in the degree of reversibility, including those completely reversible (*TFF3*, *CABYR*), partially reversible (*MUC5AC*) and irreversible (*GSK3B*) expression changes upon smoking cessation. We have validated the SAGE expression data for *TFF3*, *CABYR*, *MUC5AC*, *GSK3B *and *ENTPD8 *using a secondary cohort of current, former and never smokers. This is the first study demonstrating smoking-induced expression changes for this particular set of genes and importantly, it is the first time partial reversibility (*MUC5AC*) and irreversibility (*GSK3B*) and has been demonstrated using two different cohorts of samples with two independent assays for expression quantification. By comprehensively identifying gene expression changes that are reversible upon smoking cessation, we have introduced genes which may in future studies be investigated for polymorphisms, as those genes which are not sufficiently induced in response to smoking may identify candidate loci of susceptibility. Similarly, those genes and functions which do not revert to normal levels upon smoking cessation may also provide insight into why former smokers still maintain a risk of developing lung cancer.

## Methods

### Specimen collection

Bronchial epithelial cells were collected by bronchial brushings from 24 subjects – 9 current smokers, 11 former smokers and 4 never smokers summarized in Table 1 – by bronchial brushing as described previously [54,55]. The subjects were volunteer smokers recruited from the community as part of a NCI-sponsored chemoprevention trial. The inclusion criteria were: age > 45 years of age and a smoking history of ≥ 30 pack years. A former smoker was defined as one who had stopped smoking for at least one year or more. None of the subjects were on bronchodilator or inhaled steroids. The samples were obtained prior to treatment with an investigational chemoprevention agent.

Brushings were obtained from the peripheral airways using a 1.8 mm brush. A table of the basic demographics of the subjects used is listed in Table 1.

### Construction of SAGE libraries

To deduce the gene expression profiles, we used a method called serial analysis of gene expression (SAGE) which quantifies gene expression by the enumeration of transcript derived sequence tags [20]. SAGE libraries were constructed from each sample using the MicroSAGE protocol [55], and sequenced to a depth of ~150,000 SAGE tags per library. SAGE libraries were deposited in NCBI GEO with accession number GSE5473. Reproducibility of SAGE libraries obtained from the same bronchial brush was shown by our group previously. The R value between two libraries from the same lysate was 0.97 [55].

### SAGE tag-to-gene mapping

Tag-to-gene mapping was performed using a combination of the May 10^th^, 2006 build of *SAGEGenie *[45]. Tags with low reliability from *SAGEGenie *in Table 2 and 3 were also cross-referenced with *TagMapper *[56].

### Statistical analysis of differentially expressed genes

Stringently, only tags which exhibited a mean tag count of ≥ 20 tags per million (TPM) in at least one of current, former or never smoker SAGE libraries were used in comparative analysis. For each specific comparison, in addition to the tag count requirement, a minimum fold change of the means of two was also required. The tag abundance requirement of a mean tag count of 20 TPM was used to filter the list of tags prior to statistical comparison to reduce the number of false positives. 8148 tags meet this criterion. Given the variability in smokers and limited sample size in this study, a non-parametric Mann Whitney U Test was used to determine if a given tag (representing a gene) was differentially expressed using a p-value threshold of p ≤ 0.05, unadjusted for multiple comparisons.

### Validation of SAGE-specific targets using quantitative RT-PCR

Select targets identified in the SAGE study were validated using quantitative RT-PCR (qRT-PCR) in a second cohort of nine current, seven former and six never smokers. Briefly, 100 ng of RNA was isolated and converted to cDNA in a 50 μl reaction volume using the High-Capacity cDNA Archive Kit (cat # 4322171, Applied Biosystems). 1 μl of the resulting cDNA was analysed by qPCR, with specified Taqman primers and TaqMan Universal PCR Master Mix (cat # 4326708), using the iCycler iQTM Real-Time PCR Detection System (Bio-Rad). *CABYR*, *TFF3, MUC5AC, GSK3B *and *Actin Beta *were monitored for 40 cycles of PCR and *ENTPD8 *for 50 cycles. Primers used for qRT-PCR are listed in Additional file 11.

## Authors' contributions

RC analyzed the SAGE and quantitative PCR data to deduce the key findings, and wrote the manuscript.

KML led the construction of all SAGE libraries and contributed to data interpretation and manuscript editing.

RTN provided insight to statistical analysis.

CM provided insight to statistical analysis as well as manuscript editing.

SL isolated the clinical samples from current, former and never smokers, and contributed to interpretation of results.

SL and WLL are the principal investigators of this project.

## Supplementary Material

Additional file 1 Supplementary Table 1 – Tags expressed in all current smoker libraries. Tags which have a raw count of greater than 2 in all 8 current smoker SAGE libraries. Click here for file

Additional file 2 Supplementary Table 2 – Tags expressed in all former smoker libraries. Tags which have a raw count of greater than 2 in all 12 former smoker SAGE libraries. Click here for file

Additional file 3 Supplementary Table 3 – Tags expressed in all never smoker libraries. Tags which have a raw count greater than 2 in all 4 never smoker SAGE libraries. Click here for file

Additional file 4 Supplementary Table 4 – Tags expressed in all 24 SAGE libraries. Tags which have a raw count greater than 2 in all 24 SAGE libraries. Click here for file

Additional file 5 Supplementary Table 5 – 609 tags differentially expressed between current and never smokers. 609 differentially expressed tags between current and never smokers. Click here for file

Additional file 6 Supplementary Table 6 – 161 tags with reversible expression upon smoking cessation. 161 tags which exhibit statistically reversible gene expression upon smoking cessation. Click here for file

Additional file 7 Supplementary Table 7 – 152 tags with irreversible expression upon smoking cessation. 152 tags which exhibit statistically irreversible gene expression upon smoking cessation. Click here for file

Additional file 8 Supplementary Table 8 – 157 tags differentially expressed between mild and moderate/severe COPD. Click here for file

Additional file 9 Supplementary Table 9 – Cycle threshold data from quantitative RT-PCR. Raw cycle threshold data for quantitative RT-PCR of 5 genes. Click here for file

Additional file 10 Supplementary Table 10 – Fold-changes and p-values from quantitative RT-PCR analysis. Data from the analysis of the quantitative RT-PCR results. Click here for file

Additional file 11 Supplementary Table 11 – Quantitative RT-PCR primers. Primers ordered from *Applied Biosystems *for 5 genes. Click here for file
